# Topical ruxolitinib for prebullous phase of immune checkpoint Inhibitor–Induced bullous pemphigoid

**DOI:** 10.1016/j.jdcr.2026.08.038

**Published:** 2026-08-28

**Authors:** Ines Lederbogen, Barbara Meier-Schiesser

**Affiliations:** a Department of Dermatology, University Hospital Zurich, Zurich, Switzerland; b Medical Faculty, University of Zurich, Zurich, Switzerland

**Keywords:** cutaneous adverse drug reaction, immune checkpoint inhibitor-induced bullous pemphigoid, immune checkpoint inhibitors, Janus kinase inhibitors, topical ruxolitinib

## Introduction

Immune checkpoint inhibitor–induced bullous pemphigoid (ICI-BP) is a rare but increasingly recognized immune-related adverse event associated with programmed cell death protein 1/programmed death-ligand 1 blockade.^1^^,^^2^ Management remains challenging, particularly in the context of ongoing antitumour therapy.

Current treatment strategies rely on corticosteroids and systemic immunosuppression, with increasing use of targeted immunomodulatory therapies.^3^ Emerging evidence implicates the JAK/signal transducer and activator of transcription signaling pathway in autoimmune bullous diseases, suggesting a potential role for JAK inhibition.^4^ We report a case of ICI-BP treated with topical ruxolitinib.

## Report of a case

A male patient in his 80s was diagnosed with a moderately differentiated cutaneous squamous cell carcinoma of the high parietal scalp. Neoadjuvant cemiplimab (350 mg intravenously every 3 weeks) was initiated. After 4 treatment cycles, surgical resection was recommended by the multidisciplinary tumor board. However, the patient declined surgery. Therefore, cemiplimab was continued for an additional 3 months with planned reassessment for surgery. Twenty days later, he developed a pruritic cutaneous eruption localized to the upper chest, arms, and upper back (Fig 1 *A*), raising suspicion of a cutaneous immune-related adverse event. Skin biopsy showed an inflammatory infiltrate consistent with a drug-induced maculopapular eruption (Fig 1, *C* and *D*). The patient was subsequently enrolled in a prospective study investigating the use of topical ruxolitinib 1.5% cream for programmed cell death protein 1 inhibitor-associated cutaneous eruption (SNCTP000005796). Topical ruxolitinib 1.5% cream was applied twice daily as a thin layer to affected areas (maximum body surface area approximately 20%). During the 12-week treatment period, a total of 4 60-g tubes were used (240 g total; average approximately 2.9 g/day), with higher use during the initial phase and decreasing application as lesions improved. Within 3 days of initiating twice-daily topical ruxolitinib, pruritus markedly improved and no new papules developed. Residual erythematous papules resolved completely within 2 weeks. During the 12-week treatment course, the patient remained free of both cutaneous eruption and pruritus (Fig 1 *B*). Four days after discontinuation of topical ruxolitinib (study completion), pruritus recurred and progressively worsened. This was followed by a recurrent erythematous papular eruption involving the upper chest and upper back, which subsequently evolved into a bullous eruption within 3 weeks. Clinical examination revealed disseminated tense bullae of varying sizes on an erythematous base, with areas of secondary impetiginization (Fig 2, *A* and *B*). In accordance with institutional guidelines, the patient was hospitalized for management of a grade 3 cutaneous immune-related adverse event. Given the ongoing immune checkpoint inhibitor therapy, intensive treatment with high-potency topical glucocorticoids was initiated instead of systemic corticosteroids. ICI therapy was discontinued. A subsequent skin biopsy showed subepidermal blister formation with numerous eosinophils within the blister cavity and at the base (Fig 2 *C* and *D*). Indirect immunofluorescence revealed a pattern consistent with bullous pemphigoid. Direct immunofluorescence demonstrated linear C3 deposition along the dermal–epidermal junction. bullous pemphigoid antigen 180 and bullous pemphigoid antigen 230 IgG autoantibodies were not elevated. Based on the compatible clinical presentation, histopathological findings, and immunofluorescence results, a diagnosis of seronegative bullous pemphigoid was considered. Despite continued intensive topical corticosteroid therapy after discharge, clinical improvement remained insufficient. As topical ruxolitinib was administered within a clinical trial and is not approved for ICI-BP, continuation of therapy was not possible. Consequently, systemic treatment with dupilumab was initiated.

**Fig 1:**
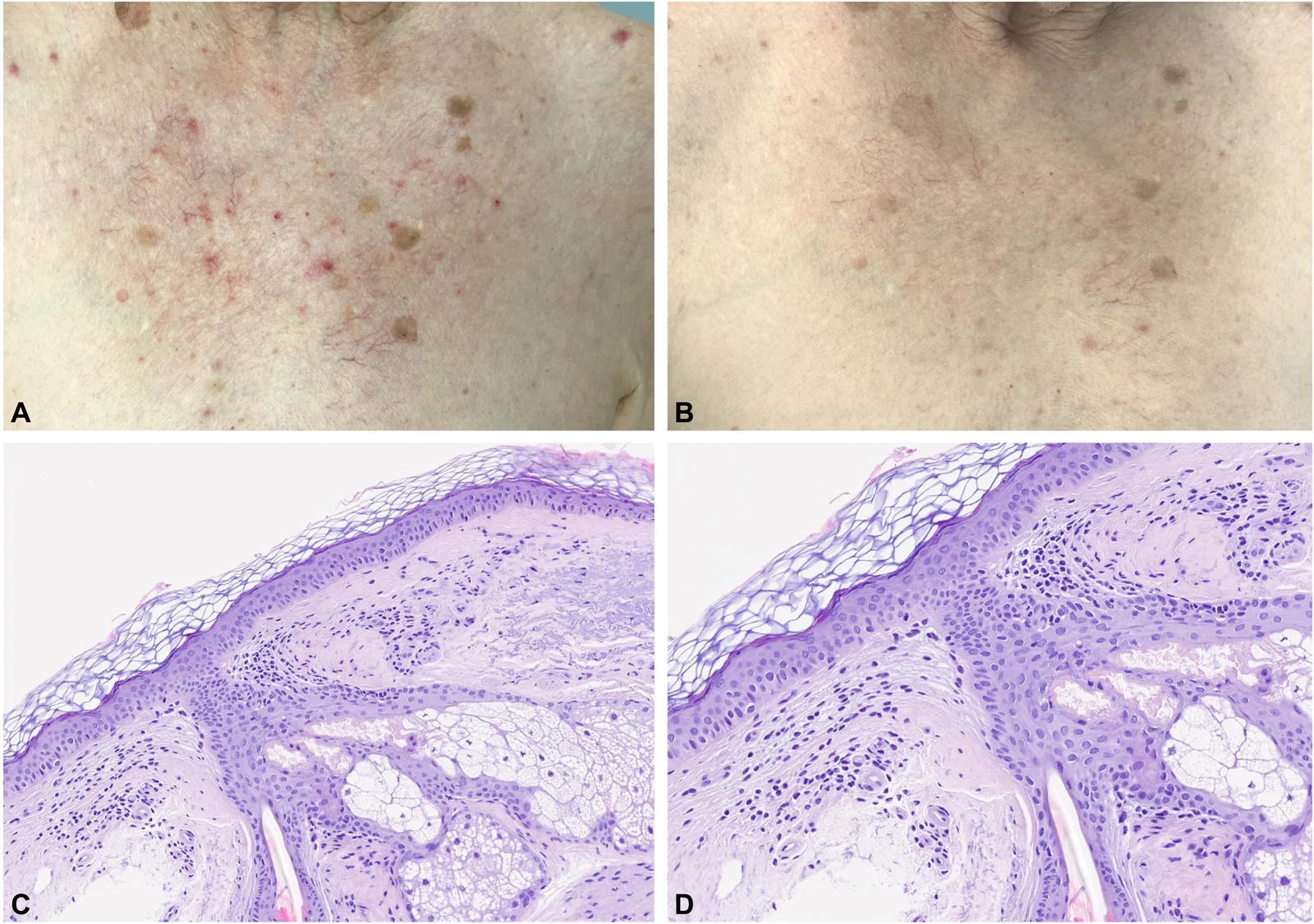
Clinical and histopathologic features of the initial immune checkpoint inhibitor–associated cutaneous eruption. **A,** Baseline lesions prior to treatment with topical ruxolitinib. **B,** Clinical improvement during treatment. **C** and **D**, Initial histopathologic findings demonstrating a discrete lymphocytic dermal inflammatory infiltrate compatible with a drug-induced maculopapular eruption. **C,** Overview. **D,** Hematoxylin and eosin stain, original magnification ×20.

**Fig 2:**
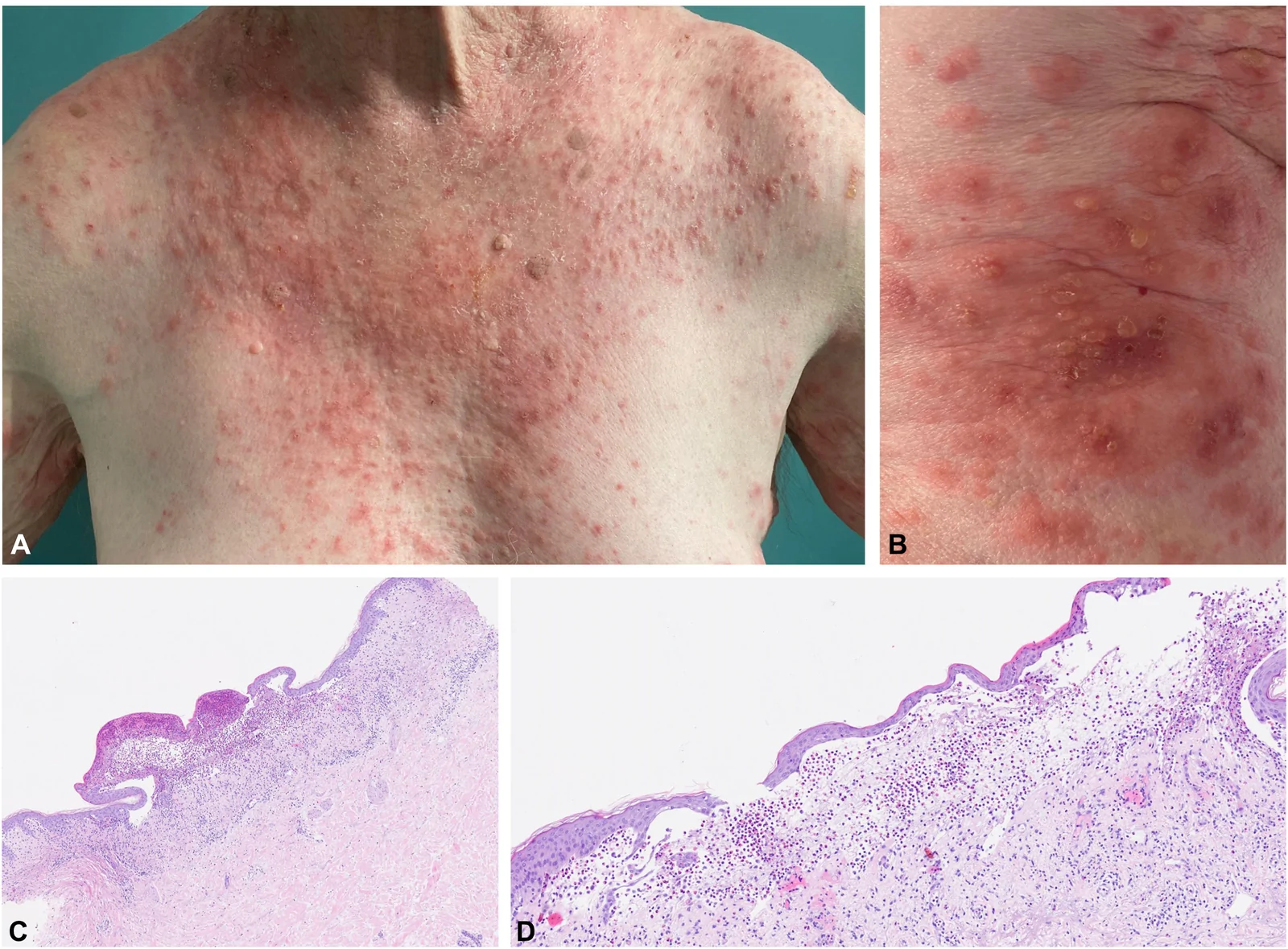
Clinical and histopathologic features of immune checkpoint inhibitor–induced bullous pemphigoid following discontinuation of topical ruxolitinib. **A** and **B,** Clinical recurrence after discontinuation of topical ruxolitinib, showing disseminated tense bullae. **C** and **D,** Histopathologic findings demonstrating subepidermal blister formation with numerous eosinophils within the blister cavity and at the dermal–epidermal junction. **C,** Overview. **D,** Hematoxylin and eosin stain, original magnification ×20.

## Discussion

This case suggests that topical ruxolitinib, a JAK1/2 inhibitor, may represent a potential treatment option for immune checkpoint inhibitor–related cutaneous adverse events, including bullous pemphigoid. In patients undergoing ICI therapy, systemic immunosuppressive treatments are often avoided due to concerns regarding interference with antitumor immunity.^1^ In this context, topical JAK inhibition may offer a targeted approach with limited systemic exposure.

Recent studies have implicated the JAK/signal transducer and activator of transcription signaling pathway in the pathophysiology of autoimmune bullous diseases, supporting the rationale for JAK inhibition in this setting.^4^ In addition, clinical reports have described improvement of bullous pemphigoid with systemic JAK inhibitors in refractory cases.^5^ However, systemic JAK inhibition may be associated with adverse events and potential risks in oncologic patients.

The observed clinical response with complete resolution of skin symptoms during topical ruxolitinib treatment and recurrence of pruritus 4 days after treatment discontinuation, followed by progression to a bullous eruption, support a potential therapeutic effect. Topical ruxolitinib was administered during the prebullous, maculopapular phase of the eruption, and no bullae were observed while the patient was on treatment. This case therefore demonstrates an effect of topical ruxolitinib on the prebullous stage of the disease rather than on established bullous pemphigoid itself, which limits the conclusions that can be drawn regarding its efficacy in treating ICI-BP directly. Further studies are needed to evaluate the efficacy and safety of topical JAK inhibitors in the management of ICI-associated bullous pemphigoid.

### Declaration of generative AI and AI-assisted technologies in the writing process

Not applicable.

## Conflicts of interest

None disclosed.
